# First report of a new corneal pathogen: *Phaeoacremonium parasiticum*

**DOI:** 10.1007/s10096-020-03980-y

**Published:** 2020-07-12

**Authors:** Horace Massa, Arnaud Riat, Georgios D. Panos

**Affiliations:** 1 Department of Ophthalmology, Geneva University Hospitals, Rue Alcide – Jentzer 22, 1211 Genève 14, CH Switzerland; 2 Service of Laboratory Medicine, Department of Diagnostic, Geneva University Hospitals and Geneva University, Geneva, Switzerland; 3 Eye Treatment Centre, Whipps Cross University Hospital, Barts Health NHS Trust, London, UK

**Keywords:** Keratitis, Eye, Fungus, Microbiology, Confocal in vivo imaging, Phaeoacremonium

## Abstract

Keratitis is a public health issue in developing countries and a potentially sight-threatening condition. Collagen fibrils in the corneal stroma are parallels to each other. Fundamental substance maintains the same space between collagen fibrils. That is how corneal transparency can be achieved. Any damage which can modify this structure will lead to corneal opacity and loss of vision. Fungal keratitis might appear in up to one-third of cases. Nevertheless, fungal keratitis remains poorly described and understood. Herein, we present the first ever reported case of corneal infection due to *Phaeoacremonium parasiticum* in a young patient. We describe the clinical and microbial characteristics, and we also discuss the use of confocal microscopy in early diagnosis of this infection.

## Introduction

Corneal-related disorders are the fifth cause of blindness worldwide [1]. Any damage which will affect the corneal anatomy will lead to corneal opacity [2]. Collagen fibrils in the corneal stroma are parallels to each other. Fundamental substance maintains the same space between each collagen fibril, that is how corneal transparency can be achieved [3].

Infectious keratitis will induce corneal scarring [4, 5]. Therefore, accurate and prompt diagnosis is of outmost importance for patient prognosis and quality of life [6].

Bacterial keratitis remains the main cause of infectious keratitis, with a prevalence over 90% in northern USA, but fungal keratitis might appear in up to one-third of cases in more southern locations of the country [7]. Moreover, fungal keratitis has a longer healing time and leads to five times more corneal perforations, which makes it a much more severe condition [8].

Appropriate diagnosis of fungal keratitis and identification of fungal isolates to species is of utmost importance as some fungi might be unresponsive to conventional antifungal treatment [9].

We, herein, present the first ever reported case of corneal infection due to *Phaeoacremonium parasiticum* in a young female patient. The patient provided written consent form.

## Case description

A 30-year-old female patient attended our eye casualty complaining of pain in the right eye.

History revealed that she was wearing monthly colored contact lenses for cosmetic purposes over the last 2 weeks. She had no history of trauma nor immunosuppression. Five days prior to her presentation, she had a consultation with her ophthalmologist in Brazil and was diagnosed with keratitis. She was discharged from the doctor’s clinic with gentamycin drops 5 times a day which improved slightly her symptoms.

Upon presentation at Geneva University Hospital, corneal sensitivity was preserved. Visual acuity was 5/10 Snellen unaided and could only be improved to 10/10 with pinhole.

At slit lamp examination, the cornea was clear except the center where small sub epithelial infiltrates were clearly visible with some fluorescein staining, but surprisingly the conjunctiva was calm.

Instead of gentamycin, the patient received a prescription of moxifloxacin drops 6 times a day.

**Fig. 1:**
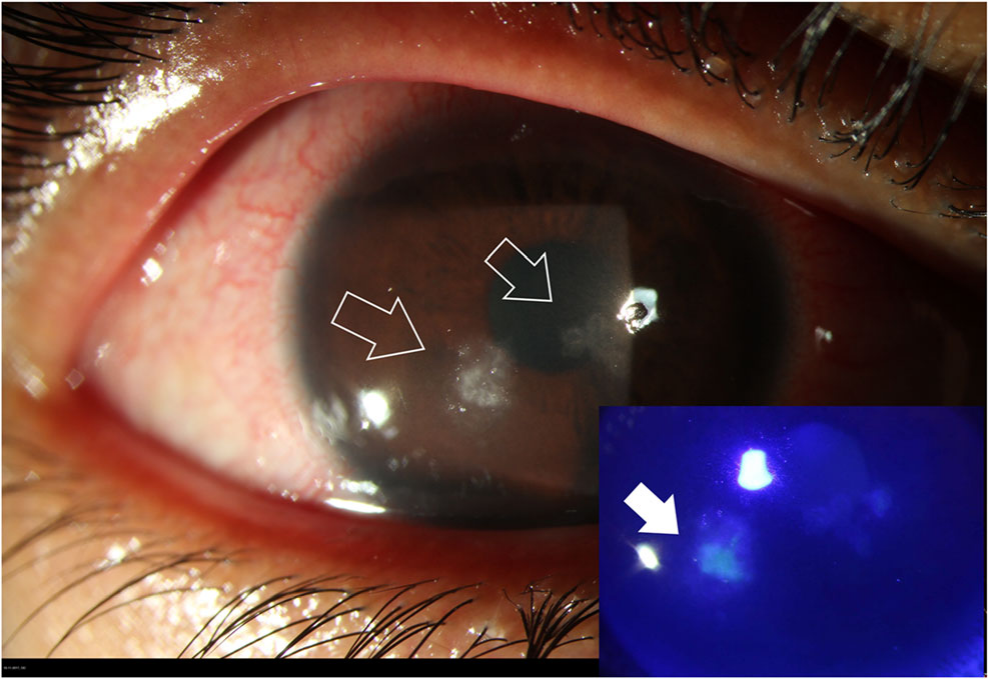
Slit lamp images of the right eye: white arrows pointing the fungal keratitis with one satellite lesion. Bottom right, the same image with fluorescein test revealing epithelium damage and the stromal impregnation of the yellowish dye (full white arrow)

Two days later, the patient came back with a deterioration of her condition. She was experiencing pain, decreased vision and photophobia (Fig. 1).

The conjunctiva was inflamed, the cornea was diffusely oedematous, with sub epithelial infiltrates staining with fluorescein and signs of inflammations (cells 0.5+, small retro descemetic precipitates 1+) were visible in the anterior chamber. Neither perineuritis nor deep stromal abscess was present.

Corneal scrapes were collected, the patient was admitted and chlorhexidine combined with desomedine local drops given hourly was initiated.

**Fig. 2:**
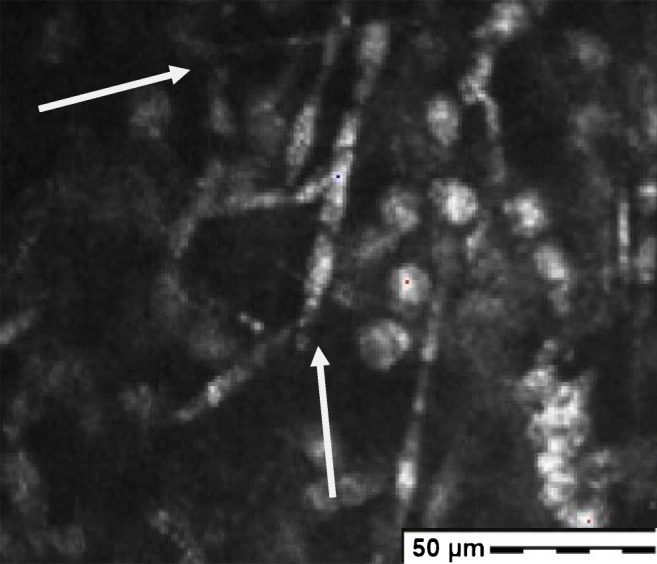
Image of the corneal lesion showing hyper reflective round structure corresponding to inflammatory cells and the presence of highly reflective fungal hyphae (white arrows) at 70 microns stromal depth (picture acquired with the Heidelberg Retina Tomograph II Rostock Cornea Module)

At day 1 after hospitalization, corneal confocal microscopy revealed the presence of filaments in the anterior stroma with no evidence of cysts (Fig. 2) and the direct microscopy examination (Fungi Fluor kit) in the laboratory confirmed the diagnosis of a filamentous fungus. The treatment was switched to liposomal amphotericin B and voriconazole drops hourly with desomedine every 2 h until the amoebic PCR came back negative after 4 days.

The patient was seen daily with daily corneal scrapes for 4 days to diminish the load of filament and to allow a better penetration of antifungal drops.

**Fig. 3:**
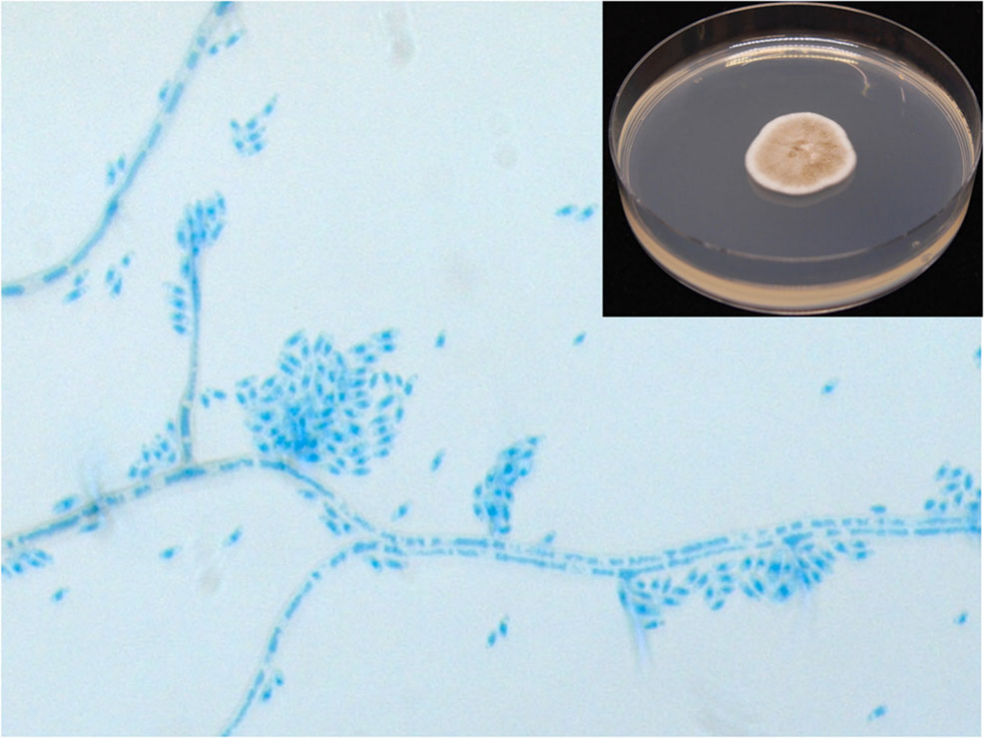
Main picture: lactophenol cotton blue stain reveals phialides bearing apical clusters of cylindrical to sausage-shaped hyaline conidia. Top left: the colonies have a moderate growth in Sabouraud agar at 30 °C; the appearance of the colonies is velvety, white-grey to brown with radial furrows

After 6 days of incubation at 30 °C, the growth of a filamentous fungus was noticeable on Sabouraud agar. The cotton blue microscopic analysis revealed germ associated with the fungus *Acremonium* sp. However, neither the microscopic nor the mass spectrometry (MALDI-TOF, Biotyper 4.1 Bruker Daltonic, Bremen, Germany) analysis could identify the pathogen. The filamentous fungus responsible for the keratitis was identified by internal transcribed spacer (ITS) sequencing as *Phaeoacremonium parasiticum* with 99.6% similarity (GenBank: AB190405.1) (Fig. 3—image filament in culture). Antifungal susceptibility testing was performed using a standardized broth microdilution method (Sensititre™ YeastOne™). The Minimal Inhibitory Concentrations (MIC) of amphotericin B, fluconazole, itraconazole, posaconazole and voriconazole are respectively 1 μg/ml, 64 μg/ml, 16 μg/ml, 0.12 μg/ml and 0.12 μg/ml.

Other laboratory tests including HIV serology, bacterial culture, herpes PCR and amibian PCR were negative.

As clinical evolution was favorable, no systemic treatment was considered. After 10 days, the patient was discharged with topical treatment of voriconazole and liposomal amphotericin B (1 drop 8 times a day for both). At day 1 after discharge, the vision was 4/10 (Snellen units) unaided and 6/10 with pinhole. The cornea had still 2 paracentral infiltrates with no more surrounding inflammatory stroma and no more staining. Anterior chamber was also quiet. At day 5 after discharge, and 15 days of topical antifungal treatment, the treatment was discontinued with no recurrence of the infection after 2 weeks.

**Fig. 4:**
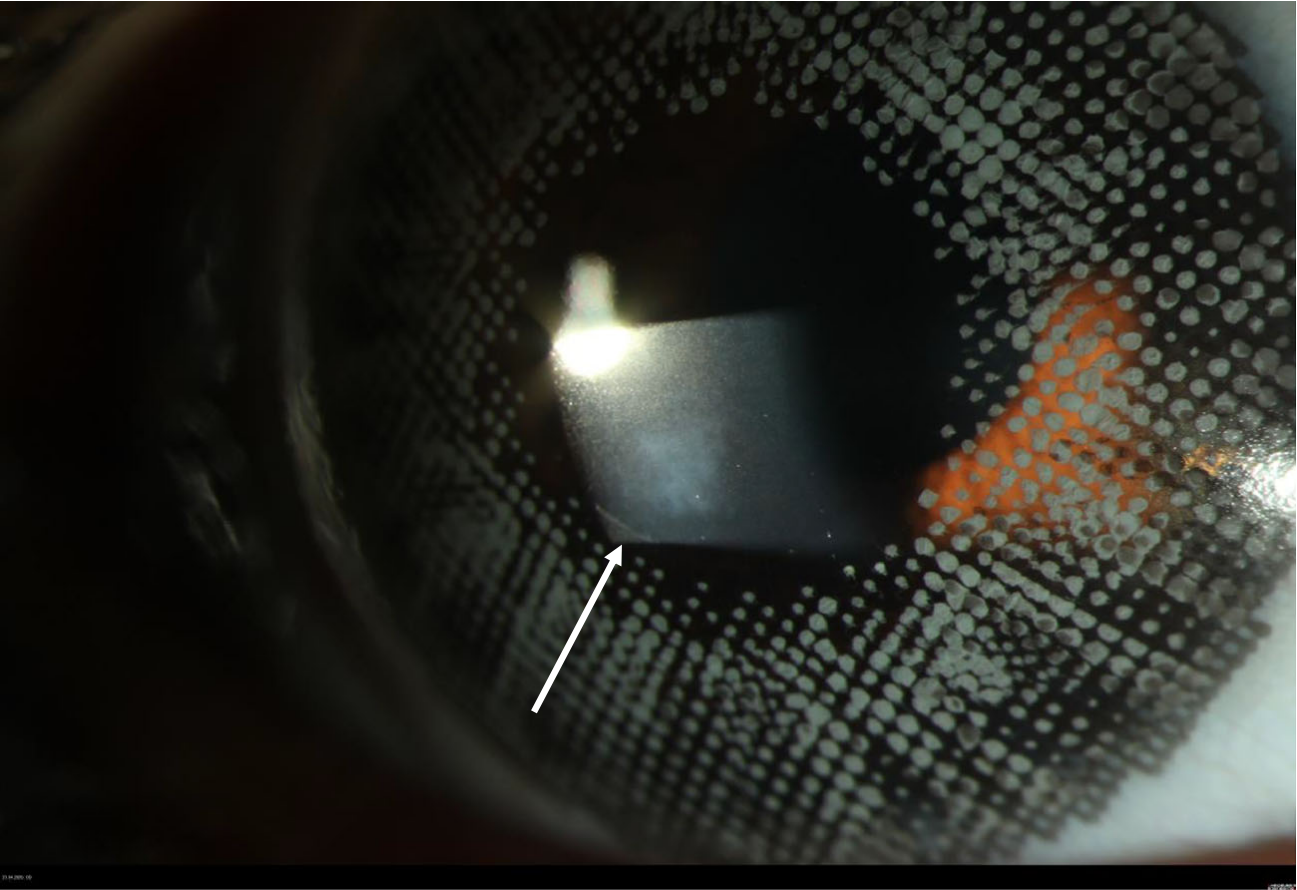
Slit lamp image of the cornea with a healthy epithelium and a sub epithelial scare (white arrow) 1 year after discharging the patient. Note that the patient is still wearing cosmetic contact lenses

At 1-year follow-up visit, visual acuity was 1.0 unaided (Fig. 4).

## Discussion

As abovementioned, it is important that the clinician is aware of the new pathogens. *Phaeoacremonium parasiticum* is a known pathogen in the agricultural domain, and especially in grapevine culture [10].

The first human case reported was in 1974 [11]. Since then, an increasing amount of human infections have been reported, especially in immunocompromised patients as they are opportunistic pathogens [12]. In ophthalmology, only one case has been reported to our knowledge, as a late-onset of post-traumatic endophthalmitis due to a phaeoacremonium [13].

Since almost all germs are unable to penetrate a healthy corneal epithelium, the history of ocular trauma should always be investigated.

In our case, the patient had two major risk factors: the patient was a contact lens wearer coming from a tropical country. As mentioned above, fungal keratitides are more frequent in tropical countries. Also fungal aetiology differs since filamentous keratitis is more frequent in tropical region, whereas in temperate countries, yeast represents the most common cause of fungal corneal infections [14]. Another important risk factor is the cosmetic contact lenses, as they have an almost 2 times increased risk of developing a keratitis compared with refractive contact lenses [15].

Clinical differentiation between a bacterial or a fungal keratitis is not obvious even for corneal specialists [16], but the failure of the initial antibiotic treatment and the progression of the lesion in the stromal cornea should make the clinician consider fungal keratitis as the differential diagnosis. In this case, two characteristics of the filament infection made the differential diagnosis difficult.

1. The slow growth of the *Phaeoacremonium parasiticum* both in culture and in the patient’s cornea is atypical for a fungus and it is a characteristic clinician should bear in mind before excluding a fungal keratitis.
2. Another characteristic is the sub epithelial spread of the disease, as on grapevine leaf, visible on OCT corneal imaging, with only little destruction of the stroma and circumscribed inflammatory reaction, whereas two-thirds of fungal keratitis usually overcome 33% depth of the stroma [17].

Confocal in vivo imaging showing filamentous infiltrate in the anterior corneal stroma helped us to consider a differential diagnosis. It is a very useful tool and easily accessible but it highly depends on the observer experience [18]. Moreover, *Phaeoacremonium parasiticum*’s hyphae are thinner than the classical *Aspergillus* hyphae making the diagnosis even more difficult, but, luckily, we had a positive direct examination and culture with the MALDi ToF.

MALDI ToF revolutionized clinical microbiology in the last 10 years and has proven its identification power not only with bacteria but also with fungi. A correct identification depends mostly on the database, which need to be as robust as diverse. The diversity is the Achilles heel of the method particularly with fungi and their vast kingdom. The latest version of the Bruker database does not contain any spectra of *Phaeoacremonium parasiticum*. In order to thwart the lack of spectra, some laboratories have developed their own database. The real improvement came from the public database proposed by Pr. Piarroux which is constituted with a large diversity of fungi including *Phaeaocremonium parasiticum* [19]. At the time of that clinical case, we had to do an ITS sequencing to be able to identify the pathogen. ITS sequencing allows a correct fungal identification in most of the fungi encounter in clinic; however, in comparison with MALDI ToF, it is expensive, time consuming and a highly specialized technician is needed. Thus, the use of a MALDI ToF public database remains a valuable option to identify uncommon clinical fungi.

The in vitro susceptibility profile of *Phaeoacremonium parasiticum* has been proposed by Badali et al. [20] and shows a similar pattern with *Phaeoacremonium parasiticum* isolated from the cornea of our patient. *Phaeoacremonium parasiticum* showed a low MIC for amphotericin B, voriconazole and posaconazole. Fluconazole and itraconazole are in contrary less active. The paucity of data concerning that fungus does not allow any implementation of breakpoint. However, clinical guidelines are proposed by the European Society of Clinical Microbiology and Infectious Disease and European Confederation of Medical Mycology [21].

Clinician and biologists should be aware of this new corneal pathogen–causing keratitis. Unusual evolution should lead to further culture investigations, confocal corneal imaging and prompt instauration of antifungal therapy to achieve the best prognosis.
